# Magnetic compression anastomosis in gastrointestinal surgery: a systematic review of early human experience

**DOI:** 10.1007/s00464-026-12956-6

**Published:** 2026-07-16

**Authors:** Arshpreet Kaur, Samreen Shaikh, Sebastian C. Valdivieso Rueda, Faran Bokhari, Vishnu R. Mani

**Affiliations:** 1 Department of Surgery, OSF Saint Francis Medical Center, 530 NE Glen Oak Ave, Peoria, IL 61637 USA; 2 University of Illinois College of Medicine at Peoria, Peoria, IL USA

**Keywords:** Magnetic compression anastomosis, Magnamosis, Anastomotic leakage, Laparoscopic, Bariatric surgery

## Abstract

**Introduction:**

Magnetic compression anastomosis (MCA) is one of the recent emerging sutureless minimally invasive techniques for maintaining the continuity of the intestine using magnetic compression. Despite its potential advantages, clinical application remains limited due to scarce evidence. The aim of this article is to review the current literature for this alternate method of bowel anastomosis.

**Methods:**

A systematic review was conducted in accordance with PRISMA 2020 guidelines. MEDLINE, Embase, and the Cochrane Library were searched from inception to 2025. Eligible studies reported MCA use in human subjects for gastrointestinal indications with at least one clinical outcome reported. Study quality was assessed using MINORS criteria for non-randomized studies and CARE guidelines for case reports.

**Results:**

Thirteen studies comprising 106 patients from nine countries were included (5 prospective first-in-human trials; 2 retrospective cohorts; 6 case reports). Clinical domains spanned adult gastrointestinal obstruction without general anesthesia, post-urological bowel reconstruction, laparoscopic pancreatoduodenectomy reconstruction, metabolic surgery, and congenital rectal atresia. Anastomotic leakage attributable to MCA was 0% (0/106) across all studies, anatomical regions, and device types. Laparoscopic MCA reduced choledochojejunostomy time from 25 to 57 min to a median of 11 min. Pediatric MCA achieved sphincter-preserving atresia repair with excellent 3-year functional outcomes.

**Conclusion:**

Early clinical evidence suggests that MCA is a safe and feasible alternative to conventional suturing or stapling techniques, with encouraging short-term outcomes. It is a promising alternative technique, especially for high-risk cases who cannot undergo general anesthesia or for those with frozen abdomens. MCA with an endoscopic technique can enable immediate resumption of oncological treatment. Due to limited clinical evidence and lack of sufficient data, further clinical trials are warranted to establish safety, efficacy, and cost-benefit before widespread human application.

**PROSPERO registration:**

1268302.

**Graphical abstract:**

**Figure:**
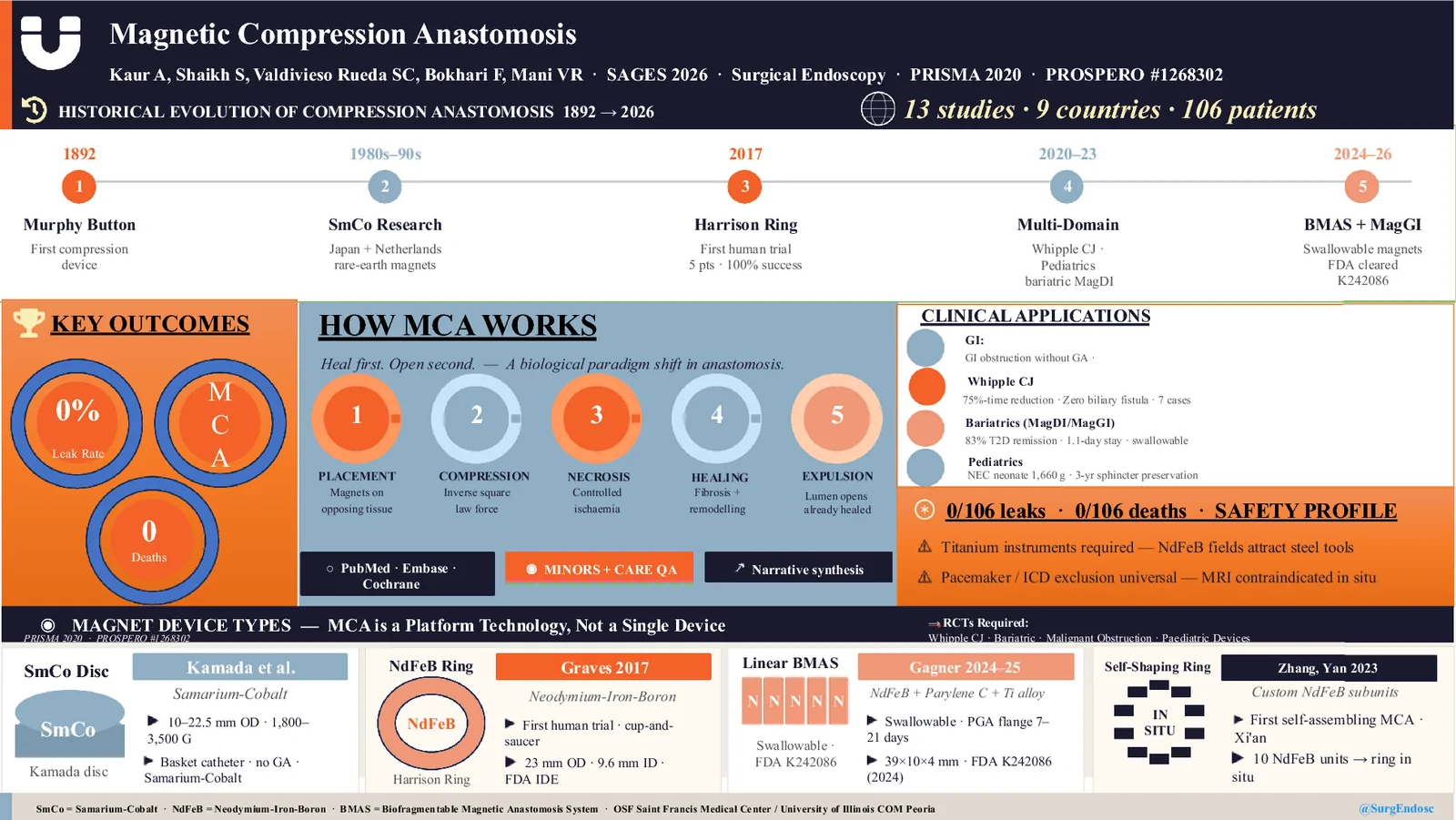

**Supplementary Information:**

The online version contains supplementary material available at https://doi.org/10.1007/s00464-026-12956-6.

The creation of reliable gastrointestinal anastomosis remains one of the most fundamental steps in abdominal surgery. For more than a century, surgeons have relied primarily on hand-sewn sutures and mechanical stapling devices to restore luminal continuity. Although these techniques are widely accepted and have undergone substantial refinement, they remain associated with limitations. Sutures and staples require tissue penetration and foreign material into the anastomotic site, which may contribute to local inflammation, ischemia, and complications such as anastomotic leakage or stricture formation. These limitations have sustained longstanding interest in alternative strategies that could simplify anastomotic construction while preserving or improving biological healing [1].

Compression-based anastomosis represents an alternative strategy for tissue approximation that avoids penetrating fixation. Sustained circumferential pressure applied between opposing tissue surfaces produces localized ischemia and necrosis of the compressed tissue layer while preserving perfusion in the surrounding tissue. Subsequent epithelial proliferation and mucosal bridging result in the formation of a continuous luminal channel. Experimental studies have demonstrated that compression anastomoses may produce favorable histologic healing characteristics, including improved epithelialization and reduced inflammatory response compared with conventional sutured anastomoses [2].

The concept of compression-mediated bowel anastomosis dates to 1826, when Felix-Nicholas Denans of Marseilles first described a sutureless end-to-end intestinal anastomosis using metallic rings [3, 4]. John Benjamin Murphy, who developed the Murphy button as a mechanical device designed to create intestinal anastomoses without sutures based on the concept of compression-mediated anastomosis [5]. Although widely used in the early twentieth century, complications including device retention, obstruction, and difficulty controlling compression forces eventually limited its adoption. Interest resurfaced in the late twentieth century with early clinical reports from the Netherlands describing magnetic rings used to create colorectal anastomoses, demonstrating that magnetic attraction could generate sufficient compressive force to approximate bowel walls [2, 5]. Subsequent experimental and clinical work conducted in Moscow during the 1980s and early 1990s further explored magnetic compression anastomosis, particularly in pediatric surgery, and included extensive animal experimentation as well as clinical applications in children with gastrointestinal and urological disorders. Collectively, these studies provided important insights into magnet geometry, compression forces, and tissue healing dynamics, emphasizing the importance of a controlled compression gradient that allows central tissue necrosis while maintaining peripheral perfusion necessary for epithelial regeneration. These studies provided important insights into magnet geometry, compression forces, and tissue healing dynamics [6]. Parallel experimental work in Japan extended the technology to vascular anastomosis, establishing key biomechanical principles regarding magnet strength and tissue interaction [7]. Modern devices commonly employ neodymium-iron-boron permanent magnets capable of generating strong magnetic forces within compact geometries suitable for minimally invasive surgical deployment. The translational work of Michael R. Harrison, who applied sustained magnetic forces to remodel biological tissues in vivo, provided the conceptual foundation for the contemporary Magnamosis device, subsequently used by Claire E. Graves and colleagues in the first modern human application of magnetic compression anastomosis for small bowel reconstruction [8].

More recently, magnetic compression anastomosis has been explored across a variety of gastrointestinal applications, including restoration of bowel continuity, management of gastrointestinal obstruction, congenital rectal atresia, hepatopancreatobiliary reconstruction, and metabolic surgery. Modern advances in magnet engineering, minimally invasive techniques, and endoscopic delivery systems have renewed interest in this technology as a potential alternative to conventional anastomosis, particularly for high-risk patients unable to tolerate general anesthesia and for complex operative scenarios. Despite increasing clinical interest, the available evidence remains limited to small case series and individual reports, and no comprehensive synthesis of human clinical data currently exists. The objective of this systematic review was therefore to synthesize the available human clinical evidence regarding magnetic compression anastomosis in gastrointestinal surgery, with particular focus on procedural feasibility, clinical outcomes, and potential complications.

## Materials and methods

### Study design

This systematic review was conducted in accordance with PRISMA 2020 guidelines. The study protocol was prospectively registered in the PROSPERO international database of systematic reviews (Internal Registration Record No: 1268302).

A systematic review of clinical literature evaluating magnetic compression anastomosis (MCA) in gastrointestinal surgery was conducted. The study aimed to summarize early human clinical experience with this technology, focusing on technical feasibility, clinical outcomes, and potential surgical applications.

### Literature search and study selection

A systematic search was performed across PubMed/MEDLINE, Embase, and the Cochrane Library from inception to May 2025. Search terms included: “magnetic compression anastomosis”, “magnamosis”, “magnetic anastomosis”, “MCA gastrointestinal”, “magnetic rings”, “magnetic gastrointestinal bypass” combined with MeSH terms for specific regions and indications.

### Eligibility criteria

Studies were eligible if they: (1) reported MCA use in human subjects for any gastrointestinal surgeries extending from the stomach to the anal canal, including pancreaticoduodenectomy. (2) Described at least one clinical outcome. Animal studies, purely preclinical reports, conference abstracts, and editorials were excluded.

### Quality assessment

Study quality was assessed using the MINORS (Methodological Index for Non-Randomized Studies) [9] criteria for cohort studies and the CARE (CAse REport) guidelines for individual case reports [10]. Full MINORS item scores for all non-case-report studies and CARE domain assessments for all case reports are reported in Supplementary Table S1. For MINORS scoring, each of the eight non-comparative items was rated 0 (not reported), 1 (reported but inadequate), or 2 (reported and adequate), yielding a maximum score of 16. For CARE assessment, six key domains were evaluated per report: clinical history and timeline, diagnostic evaluation, intervention details, follow-up and outcomes, patient perspective, and confirmation of informed consent.

### Data extraction

Two investigators independently extracted study-level data, including: author and year, study location, study design, number of patients, clinical indication, magnet type, complications, follow-up duration.

*Outcome*: The primary outcome was the anastomotic leakage rate, defined as clinical, radiological, or operative evidence of leakage from the MCA anastomosis. Secondary outcomes included: anastomosis-related mortality, procedure time, anastomosis formation time, magnet expulsion time, restenosis, perforation, and disease-specific outcomes (weight loss and type 2 diabetes [T2D] remission for bariatric studies; biliary and pancreatic fistula for laparoscopic Whipple studies).

*Statistical analysis*: This is a systematic review. No pooled statistical analysis was performed owing to the extreme heterogeneity across indications, device types, and outcome reporting standards. No comparative effect sizes, odds ratios, or confidence intervals are reported, as none of the studies employed a randomized or controlled design with a concurrent comparator group. Descriptive statistics (proportions, medians, and ranges) are reported as presented in the primary publications.

Due to the heterogeneity of study designs and outcomes, quantitative meta-analysis was not feasible.

*Ethics and consent*: This systematic review is based entirely on previously published data. No individual patient data were collected. All primary studies reported institutional ethics committee approval or waiver and patient informed consent as stated in the respective publications.

Sex-based reporting: Patient sex is reported for all studies where available. In Kamada et al. [11], 9 of 14 patients were male and 5 were female. In Graves et al. [8], 4 were male and 1 was female. In Li et al. [12], 6 were male and 1 was female. In Gagner et al. [13], 6 were male and 3 were female. In Gagner et al. MagGI study [14], 2 were male and 5 were female. In the Gagner et al. multicenter study [15], 88.0% (38/43) were female. In the Gagner et al. BMAS solo MagDI study [16], 8 of 15 (53.3%) were female. All pediatric and case-report patients were individually identified by sex in the primary publications. Sex-stratified outcome analysis was not feasible given small sample sizes across all included studies.

## Results

*Study characteristics*: A total of 13 studies met the inclusion criteria, encompassing 106 patients treated with magnetic compression anastomosis. The included studies consisted of five prospective first-in-human trials, two retrospective cohort studies and six case reports. The studies were conducted across nine countries, including the United States, Japan, China, United Kingdom, Canada, Georgia, Belgium, Czech Republic and Spain till 2025.

### Quality assessment

MINORS scores for the seven cohort and prospective studies ranged from 9 to 12 out of a possible 16 (Supplementary Table S1), consistent with moderate methodological quality across the cohort. The CARE assessment of the six case reports revealed adequate reporting across most domains, including clinical history and timeline, intervention details, and confirmation of informed consent (Supplementary Table S1).

### Clinical indications

Magnetic compression anastomosis was applied in a variety of gastrointestinal surgical settings, including gastrointestinal obstruction, colorectal stenosis, congenital rectal atresia, pancreaticobiliary reconstruction, metabolic surgery demonstrating broad applicability of MCA across multiple surgical domains.

Study characteristics are summarized in Table 1.

**Table 1:** Study characteristics

Study (Year)	Institution, country	Design	N	Primary indication
Graves et al. [8]	UCSF, San Francisco, USA	Prospective FIH	5	Small bowel anastomosis after ileal urinary diversion
Kamada et al. [17]	IUHW Hospital, Japan	Case report	1	Sigmoid anastomotic stenosis—side-to-side MCA
Liu et al. [18]	NW Women and Children Hospital/Xian JTU, China	Case report	1	Rectal atresia following NEC; premature neonate
Kamada et al. [11]	IUHW Hospital, Japan	Retrospective cohort	14	GI obstruction/stenosis across all regions; intravenous sedation without general anesthesia
Li et al. [12]	First Affiliated Hospital, Xian JTU, China	Retrospective cohort	7	Laparoscopic pancreatoduodenectomy (LMC-CJ all 7; LMC-PJ 2 of 7)
Lu et al. [19]	First Affiliated Hospital, Xian JTU, China	E-video case report	1	Rectal stenosis
Zhang et al. [20]	First Affiliated Hospital, Xian JTU, China	E-video case report	1	Long severe eccentric colonic stenosis; novel self-shaping modular ring assembled in situ
Gagner et al. [13]	Czech Republic/Westmount, Canada	Prospective FIH	9	Primary bariatric MagJI bipartition for T2D and obesity
Gagner et al. [14, 16] MagGI	Westmount Square Surgical Center, Canada	Prospective FIH	7	Bariatric MagGI bipartition after sleeve gastrectomy
Tyraskis et al. [21]	Kings College Hospital London/Birmingham Women and Children Hospital, UK	Case report	1	Congenital high rectal atresia in a toddler
Zhang et al. [20]	First Affiliated Hospital, Xian JTU, China	E-video case report	1	Colonic stenosis
Gagner et al. [15]	Westmount (Canada)/CHU St-Pierre (Belgium)/Hospital Clinico San Carlos (Spain)/Innova Medical Center (Georgia)	Prospective multicenter	43	Bariatric MagDI + / − concurrent or prior sleeve gastrectomy (4 centers, 2 treatment groups)
Gagner et al. BMAS [16]	Westmount (Canada)/Innova Medical Center (Georgia)	Prospective FIH	15	Solo MagDI—mild obesity (BMI 30–35 kg/m2) + T2D; swallowable biofragmentable BMAS device

*FIH* first-in-human, *IUHW* International University of Health and Welfare, *JTU* Jiaotong University, *NEC* necrotizing enterocolitis, *LMC-CJ* laparoscopic magnetic choledochojejunostomy, *LMC-PJ* laparoscopic magnetic pancreatojejunostomy, *MagJI* magnetic jejunoileostomy, *MagGI* magnetic gastroileostomy, *MagDI* magnetic duodenoileostomy, *BMAS* biofragmentable magnetic anastomosis system, *T2D* type 2 diabetes mellitus, *MCA* magnetic compression anastomosis

### Device characteristics

At least eight distinct device architectures were employed across the included studies, reflecting the rapid engineering evolution of MCA from bare disc magnets to FDA-cleared biofragmentable swallowable systems and showing the absence of a single standardized MCA platform. Device characteristics are summarized in Table 2.

**Table 2:** Device characteristics

Device/study	Material	Shape	Size	Strength	Key engineering feature
SmCo discs [11]	Samarium-cobalt	Disc	10–22.5 mm OD; 5 mm thick	1800–3500 G	Bare disc; lithotripsy basket catheter delivery; sized to bowel diameter; no general anesthesia required; daughter (oral/proximal) and parent (anal/distal) convention
Harrison Rings [8]	NdFeB in polycarbonate shell (Magnomosis device)	Ring	23 mm OD; 9.6 mm ID; 6.6 mm thick	Not stated	Cup-and-saucer self-centering geometry; polycarbonate casing prevents direct tissue contact with rare-earth alloy; medical-grade biocompatible barrier; cautery patency hole
Custom Nd–Fe–P rings [18]	Nd–Fe–P low-toxicity alloy	Ring with central hole	12 mm OD; 6 mm thick	2,500 G	Nd-Fe–P clinical use;8Fballoon catheter; neonatal scale;
NdFeB + BEMD [12]	NdFeB N45; Ni + TiN composite coating	Ring + handle	9–12 mm CJ; 5–6 mm PJ	Not stated	TiN and nickel coating provides hard, chemically inert surface resistant to bile acid corrosion and ion release; BEMD rotating handle enables laparoscopic delivery; 8F bile drainage tube integrated
Linear BMAS [13, 15, 16]	NdFeB core; parylene C coating; Ti-6Al-4 V housing; PGA biofragmentable flange	Linear bar	39 × 10.2 × 4.4 mm (reduces to 38.1 × 7.6 mm on fragmentation)	Not stated	Multi-layer protection: parylene C conformal barrier + Ti alloy housing + outer flange; swallowable; flange dissolves 7–21 days; FDA cleared K242086 (2024)
Tripartite LMAS [14]	NdFeB core; parylene C; Ti-6Al-4 V ends; flanges	Tripartite linear bar	80 mm revised to 70 mm	Not stated	Fragments into 3 separate pieces on PGA dissolution for reliable expulsion; gastric antrum to ileum; pylorus navigation
Self-shaping modular ring [14]	Custom NdFeB subunits	10 units assemble into ring in situ	Variable	Not stated	First in situ self-assembling MCA ring; delivered as linear chain along guidewire through stenosis; solves the fundamental constraint that pre-formed ring diameter must exceed stenosis diameter
NdFeB rings N42/N52 [17]	NdFeB N42 (2nd attempt); N52 (1st attempt)	Ring with central guidewire hole	10 mm dia 5mm thick, (1st); 15 mm diameter, 2 mm thick (2nd)	Nd52/Nd42	Pediatric ad hoc device;N52 too strong causing premature coupling—switched to N42; guidewire through central hole anchors magnet precisely

*SmCo* samarium-cobalt, *NdFeB* neodymium-iron-boron, *Nd-Fe–P* neodymium-iron-phosphorus, *OD* outer diameter, *ID* inner diameter, *BEMD* bilioenteric magnamosis device, *BMAS* biofragmentable magnet anastomosis system, *LMAS* linear magnet anastomosis system, *PGA* polyglycolic acid, *TiN* titanium nitride, *GA* general anesthesia, *FDA* US Food and Drug Administration, *Ni* nickel, *8F* 8 French

Magnet placement techniques varied depending on the clinical indication and included endoscopic placement, laparoscopic placement, combined endoscopic-surgical approaches, swallowable magnetic systems for metabolic bypass procedures as shown in Table 3.

**Table 3:** Magnet placement techniques along with type of anastomosis

Study	Access	Placement technique	Guidance	Anastomosis	Expulsion
Graves [8]	Open surgical	Daughter (proximal ileum); mother (distal ileum) placed intra-luminally	Direct visualization	Entero enteric channel via gradual compression necrosis	Spontaneous; 17–85 days
Kamada [17]	Endoscopic	Daughter (oral-side disc) via colostomy; mother (anal-side) via colonoscope across stenosis	Endoscopic + fluoroscopic	Accidental side-to-side colonic anastomosis in 5 days	Spontaneous day 5
Liu [18]	Endoscopic + balloon catheter	Mother ring via mucous fistula (cecal side); daughter ring via anus; 8F balloon catheter as shared guidewire rail	Endoscopic + fluoroscopic	Cecum-to-rectum channel; ileocecal valve preserved	Spontaneous day 9
Kamada [11]	Endoscopic	Daughter (oral-side) disc delivered by LBC via ileus tube or stoma; mother (anal-side) disc via colonoscope; external magnet assists distal delivery	Endoscopic + fluoroscopic	Luminal recanalization; all GI regions; mean 13 days formation	Spontaneous; mean 13 days
Li et al. [12]	Laparoscopic (BEMD)	Daughter ring into bile duct stump; BEMD handle delivers mother through intestinal wall laparoscopically	Laparoscopic visualization	LMC-CJ median 11 min; LMC-PJ 12–15 min	Device detaches; median 50 days
Lu et al. [19]	Endoscopic retrograde	Enteroscope delivers ring to proximal atretic face; ring manually inserted from anus to distal face	Endoscopic + fluoroscopic	Atresia managed with MCA + stent	Spontaneous day 13
Zhang [20]	Endoscopic	10 modular daughter units along guidewire to proximal colon—self-assemble into ring in situ; double-ring mother via colonoscope	Endoscopic + X-ray	Self-shaping ring produces colonic anastomosis in 7 days	expelled on day 7
Gagner, Fried [13]	Laparoscopic + swallowable	Daughter (distal ileal) BMAS magnet swallowed or delivered endoscopically; laparoscopic external magnetic positioning device surfs to target ileum; mother (proximal jejunal) via gastroscope	Laparoscopic + fluoroscopic + endoscopic	Jejuno-ileostomy bipartition	Biofragmentable; median 19 days
Gagner [14] MagGI	Laparoscopic + endoscopic	Daughter (distal ileal) via orogastric catheter + laparoscopic surfing; mother (proximal gastric antrum) via gastroscope	Laparoscopic + endoscopic + fluoroscopic	Gastro-ileostomy bipartition	Tripartite fragmentation; median 27 days
Tyraskis, [21]	Bilateral endoscopic	Ring with central hole threaded over guidewire anchored through atretic membrane from each side; bilateral endoscopy ensures precise placement	Bilateral endoscopy + fluoroscopy + guidewire	Rectal continuity in congenital atresia; 1st attempt failed (N52 too strong); 2nd attempt successful	Spontaneous day 5
Zhang et al [22]	Endoscopic + Zebra guidewire	Daughter (side-hole) advanced along Zebra guidewire via ileostomy; multiple mother magnets via colonoscope	Endoscopic + fluoroscopic (AP + lateral views)	Post-cancer rectal atresia recanalized; macroscopic necrotic tissue confirmed on separation	Active retrieval day 14
Gagner [15] multicenter	Laparoscopic + endoscopic	Daughter (distal ileal) delivered endoscopically; external magnetic positioning device surfs daughter through jejunum to ileum; mother (proximal duodenal) via gastroscope	Laparoscopic + fluoroscopic + endoscopic	MagDI + / − sleeve gastrectomy; 100% feasibility in 43/43	Spontaneous; median 35 d (after-SG) or 48.5 d (+ SG)
Gagner [16] BMAS	Laparoscopic + swallowable	Daughter (distal ileal) BMAS magnet swallowed in 9/15 cases or delivered endoscopically; surfed laparoscopically to ileum; mother (duodenal) via gastroscope	Laparoscopic + fluoroscopic + endoscopic	Solo MagDI without sleeve gastrectomy; 15/15 (100%)	PGA biofragmentation; median 25 days

### Primary outcome

Anastomotic leakage occurred in 0/106 patients (0%) across all 13 studies, all device platforms, anatomical regions, and indications. This finding was consistent regardless of study design, patient age, delivery technique, or geographical setting. Zero anastomosis-related deaths were recorded in any of the included studies. 

This consistency was maintained across GI regions including esophagus, stomach, small intestine, large intestine, bile duct, and pancreatic duct, and across patient populations ranging from extreme prematurity (30 + 6 weeks) to elderly high-comorbidity adults (ASA 4, age 82). Where direct comparisons were possible, MCA consistently reduced procedure time: laparoscopic choledochojejunostomy was completed in a median of 11 min (range 8–16) versus 25–57 min for conventional laparoscopic technique; pancreatojejunostomy was completed in 12–15 min versus a median of 45 min for manual suture at the same institution.

Anastomosis formation time had a mean of 13 days (range 5–24) in the Kamada [11] adult GI obstruction series and 19–27 days in the bariatric series. Operative outcomes are summarized in Table 4.

**Table 4:** Operative outcomes along with time to anastomosis formation, magnet expulsion, hospital stay and follow-up

Study	N	Procedure time	Formation (days)	Expulsion (days)	Hospital stay	Follow-up	Leak
Graves [8]	5	25–35 min	18–56	17–85(median 50)	18–51 days	Median 13 mo	0/5
Kamada [17]	1	NR	5 (side-to-side)	5	NR	Short-term	0/1
Liu [18]	1	NR	~ 9 (expelled)	9	NR	12 mo	0/1
Kamada [11]	14	Mean 44 min	Mean 13 (5–24)	Mean 13	Mean 36 days (14–120)	Mean 34 mo	0/14
Li [12]	7	Total 340 min	Median 50 d	Median 50	Median 18 days	Median 11 mo	0/7
Lu [19]	1	90 min	13	13	NR	7 mo	0/1
Zhang et al. [20]	1	NR	7	7	NR	9 mo	0/1
Gagner, Fried [13]	9	Mean 104 min	Median 19	Median 19	Mean 4.3 days (4–6)	6 mo	0/9
Gagner [14] MagGI	7	Mean 64 min	Median 27	Median 27	Mean 1.1 days (1–2)	6 mo	0/7
Tyraskis [21]	1	NR	5	5	1 day	3 years	0/1
Zhang et al. [22]	1	NR	14	14 (active removal)	NR	3 mo	0/1
Gagner [15] multicenter	43	67 min (after-SG); 175 min (+ SG)	~ 14–48	Median 35 (after-SG); median 48.5 (+ SG)	Median 1 d (after-SG); median 3.5 d (+ SG)	6–12 mo	0/43
Gagner [16] BMAS	15	Mean 51 min	7–21	Median 25 (15–29)	Mean 1.0 day	12 mo	0/15

TOTAL: 106 patients—Technical success 106/106 (100%)—Anastomotic leakage 0/106 (0%)—Zero anastomosis-related deaths

*NR* not reported, *mo* months, *d* days, *SG* sleeve gastrectomy, after-SG = MagDI as revisional second step after prior SG; + SG = MagDI with concurrent SG

### Safety and complications

There were no anastomotic leaks reported for the patients in all the studies (0%). Anastomotic restenosis occurred in 2/14 patients (14%) in the Kamada [11] series, managed with balloon dilation. One anastomotic perforation occurred (1/14; 7%) after the balloon dilation, requiring emergency surgery but with uneventful recovery. The first MCA attempt for congenital rectal atresia failed due to premature magnet coupling caused by angular mismatch between atretic pouches combined with excessively strong N52 magnets. Later, a successful second attempt was achieved with weaker N42 magnets and bilateral endoscopic guidewire guidance. In Kamada et al., the most reported complication was anastomotic restenosis, which occurred in 2/14 of patients and was generally managed with endoscopic balloon dilation. No mortality related to magnetic compression anastomosis was reported. Table 5 summarizes MCA-specific complications across all studies. Table 6 summarizes complications seen in metabolic surgery series not related to magnetic compression devices.

**Table 5:** Summarizes MCA-specific complications across all studies

Complication	N	Rate (%)	Study	Management
Anastomotic leakage	0/106	0	All 13 studies	None required
Anastomosis-related mortality	0/106	0	All 13 studies	None
Anastomotic restenosis	2/14	14	Kamada [11]	Balloon dilatation (up to 8 sessions); 1 perforation after 8^th^ dilation requiring emergency laparotomy + ileostomy (recovered, discharged day 120)
Anastomotic perforation (post-dilatation)	2 cases	–	Kamada [11]; Gagner [16] BMAS	1: emergency laparotomy (Kamada 2021); 1: late endoscopic dilation, conservative management, resolved without sequelae (BMAS 2025)
Re stenosis (post-cancer)	1/1	–	Lu [19]	Second MCA procedure + metallic stent; stoma closure achieved; normal defecation restored
Failed 1^st^ attempt (pediatric)	1/1	–	Tyraskis [21]	N52 magnets too strong; premature coupling from angular mismatch; no permanent harm; 2^nd^ attempt with N42 + guidewire succeeded
Serosal tears (intraoperative)	7/43	16	Gagner [15] multicenter	Related to laparoscopic bowel forceps grasp-and-pull during external magnetic surfing of daughter magnet; all repaired intraoperatively without sequelae; not device-related; no anastomotic consequences

*CD* Clavien-Dindo, *MCA* magnetic compression anastomosis, *BMAS* biofragmentable magnetic anastomosis system

**Table 6:** Complications related to metabolic surgeries (non-MCA device-related)

Complication	N	Rate (%)	Study	Management/Notes
CD-III SAEs (bariatric, non-device-related)	3/15	20	Gagner [16] BMAS	Duodenitis post-expulsion (IV antibiotics, resolved); hypovolemia/hypokalemia (IV electrolytes, resolved); late anastomotic perforation from endoscopic dilation (conservative, resolved)
CD-III SAEs (multicenter, procedure-related)	3/43	7	Gagner [15] multicenter	UTI prolonging hospitalization; pelvic fluid collection (transvaginal drainage); jejunal obstruction at mesenteric defect (laparoscopic repair)
Nutritional complications	–	–	Gagner MagGI [14]; Gagner, Fried [13]	Hemoglobin significantly reduced at90 days (*p* < 0.05) in MagGI; 1 patient hospitalized for hypoproteinemia; anemia in 2/7 (MagGI); monitoring + supplementation required

*CD* Clavien-Dindo, *SAE* serious adverse event, *UTI* urinary tract infection, *MagGI* magnetic gastroileostomy, *BMAS* biofragmentable magnetic anastomosis system

## Discussion

In present literature, MCA has demonstrated success in all indications, anatomical regions, and patient populations. No anastomotic leakage was seen in any MCA anastomosis in any study, which is a remarkably consistent safety signal considering that conventional anastomotic leak can result in pancreatic fistula rates of up to 50% following pancreatoduodenectomy and 30-day mortality of up to 22% following colorectal surgery. MCA makes possible a number of clinical advancements that are not possible with traditional anastomotic techniques, such as sphincter-preserving pediatric rectal atresia repair, may allow prompt post-procedure oncological treatment, swallowable device delivery, and anastomosis without general anesthesia.

Conventional anastomoses create immediate full-thickness wall penetration, relying on suture or staple material to seal a junction immediately subject to luminal pressure and bacterial colonization. From a biomechanical standpoint, magnetic compression anastomosis demonstrates superior early anastomotic strength, with ex vivo data showing significantly higher burst pressures compared to hand-sewn (47.45 mmHg), stapled techniques (38.96–70.75 mmHg) and approaching native tissue strength (162.64 vs. 181.05 mmHg) within 60 min of compression [23]. Progressive magnetic compression induces transmural necrosis at the contact zone while peripheral fibrosis forms before the channel opens. Paired magnets generate sustained circumferential compression across opposing tissue surfaces, producing localized ischemia and controlled necrosis at the contact interface while maintaining perfusion in the surrounding tissue. As tissue thins, the attractive force increases exponentially (inverse-square law), self-reinforcing the compressive seal. A mature fibrous anastomotic collar has already formed by the time the necrotic core separates [23, 24].

The prospective multicenter MagDI trial [15] enrolled 43 patients across four centers in Canada, Belgium, Spain, and Georgia, representing the largest and most geographically diverse MCA cohort to date. Seven serosal tears occurred during the grasp-and-pull maneuver performed with laparoscopic bowel forceps to assist the transit of the intraluminal daughter magnet from the proximal jejunum to the distal ileum. All the events took place during the delivery phase prior to the development of an anastomosis and were unrelated to the MCA device or anastomosis. However, these occurrences constitute a significant procedural risk unique to this method, which surgeons using the MagDI technique need to be prepared to anticipate and manage.

Kamada et al. [11] remain unique in demonstrating MCA delivery under intravenous sedation alone (0.4 mg flunitrazepam) across all GI regions in 14 patients, 79% of whom were ASA class 3 or 4. Conventional palliative bypass for malignant obstruction requires general anesthesia and 7–14 days of recovery, collectively delaying chemotherapy by 3–6 weeks. In this setting, MCA offers the possibility of minimally invasive luminal reconstruction with less physiological insult. Kamada et al. explicitly reported that MCA may allow prompt chemotherapy to be initiated almost immediately after the procedure without a recovery interval. A prospective trial measuring time-to-chemotherapy initiation comparing MCA under sedation versus conventional palliative bypass would provide the definitive evidence needed to establish this as a standard-of-care indication.

MCA also appears promising in laparoscopic pancreatoduodenectomy, where biliary and pancreatic reconstruction remain among the most technically demanding steps in abdominal surgery. In the Li et al. [12] laparoscopic pancreatoduodenectomy series, no leakage occurred from any LMC-CJ or LMC-PJ anastomosis. MCA choledochojejunostomy was completed in a median of 11 min versus 25–57 min conventionally, with zero biliary fistulas in all seven cases. MCA-PJ was done in cases where the pancreatic duct measured 5 mm or above. Absence of biliary contamination may reduce the severity of concurrent pancreatic fistulas by preventing bile-mediated activation of pancreatic enzymes in the peritoneal cavity. Although the sample size remains small, these findings suggest that MCA may simplify reconstructive steps that are otherwise technically challenging and time-consuming during minimally invasive pancreatic surgery.

Recent research has also extended MCA into metabolic and bariatric surgery [13–16], where the technology is employed to intentionally generate intestinal bypass rather than alleviate obstruction. The viability of swallowable or minimally invasive biofragmentable magnetic devices for gastroileal or jejunoileal bipartition was shown by the MagJI and MagGI experiments. The MagJI et al. [13] achieved HbA1c reduction from 7.1 to 6.1% (*p* < 0.01) at 6 months with 83% T2D remission, confirming an enteroendocrine mechanism (GLP-1 and PYY release from distal ileal L-cells) rather than caloric restriction. The 1.1-day hospital stay in the revisional MagGI et al. [13] represents a procedural simplicity that the stapled single-anastomosis sleeve-ileal bypass cannot match. At the same time, these procedures carry the same biological nutritional risks as other intestinal bypass operations. The anemia, hypoalbuminemia, and hypoproteinemia reported in some patients emphasize that the metabolic promise of MCA must be balanced against the need for careful postoperative nutritional surveillance.

MCA has been applied across the full pediatric age spectrum. A premature neonate underwent caeca-rectal anastomosis with no open dissection according to Liu et al. [18]. Another case, Tyraskis et al. [21], succeeded in congenital rectal atresia repair in a toddler with 3-year follow-up, the longest outcome in our evaluation, with excellent anorectal function, and demonstrated that magnetic devices can restore luminal continuity without open pelvic dissection and with preservation of anorectal function. These pediatric cases also highlight an important technical principle related to the fact that magnet strength matters. Excessive magnetic force may lead to premature or misdirected coupling, whereas more carefully calibrated devices combined with guidewire-assisted deployment can achieve controlled and anatomically precise compression. This suggests that future pediatric applications will likely depend on dedicated, force-calibrated devices rather than simply adapting adult systems.

Two practical safety considerations were identified. The first, arising from Graves et al.’s [8] initial case, encouraged the surgical team to switch entirely to titanium (non-magnetic) instruments from the second case onwards. Titanium or other non-ferromagnetic instrument sets should be considered a prerequisite for surgical MCA procedures. Secondly, four studies that specified the eligibility criteria, [8, 14–16] explicitly excluded patients with pacemakers or implantable cardioverter-defibrillators. The rationale is twofold: MRI is contraindicated while magnets remain in situ (patients must wear a medical alert bracelet until device passage is confirmed).

The magnetic device engineering literature further demonstrates that MCA is a family of methodologies rather than a single platform. Samarium-cobalt discs, self-centering ring magnets, bespoke rings, laparoscopic biliary handles, self-assembling modular systems and swallowable biofragmentable linear magnets were among the devices. In the Kamada series [11, 17], the oral-side (proximal) magnet is designated as the ‘daughter’ and the anal-side (distal) magnet the ‘parent’ or ‘mother’. The US Food and Drug Administration granted 510 (k) clearance (K242086) to the linear magnetic anastomosis system (LMAS) in 2024 [13], representing the first regulatory approval for a linear magnetic anastomosis device. In the Whipple series [12], the daughter (smaller ring) is introduced into the bile duct or pancreatic duct stump on the hepatic or pancreatic side, while the mother (larger ring with handle) is delivered to the bowel lumen on the enteric side. In the bariatric BMAS and LMAS series [13–16], the daughter (distal ileal) magnet is delivered first and surfed to the target position, while the mother (proximal duodenal or jejunal) magnet is positioned endoscopically. Positioning the smaller, lighter daughter proximal and the larger, heavier mother distal deliberately exploits gravitational mechanics to facilitate expulsion. This variety illustrates the field’s adaptability as well as its immaturity in the field.

While magnetic compression can be tailored to a wide range of anatomical problems, it is challenging to compare different research due to the absence of standardized equipment and magnetic force values. Future multicenter trials will require standardization of device reporting, including magnet material, size, configuration, force characteristics, and delivery mode.

Beyond the GI tract, detachable magnetic ring-assisted hepatic vascular reconstruction has reduced liver transplant anhepatic phase from 35–55 min to 9–13 min in Chinese clinical series without increasing vascular complications [7]. Development of biodegradable nanomagnetic materials that maintain adequate field strength during anastomosis formation and then degrade on healing completion is the critical next engineering step for full vascular clinical translation [7].

### Future directions

The crucial next step is to conduct randomized controlled studies contrasting MCA with traditional anastomosis. A MagJI/MagGI versus stapled single-anastomosis sleeve-ileal bypass trial with a 12-month structured nutritional follow-up, an MCA versus conventional laparoscopic choledochojejunostomy trial in pancreatoduodenectomy and a time-to-chemotherapy trial comparing MCA under sedation versus conventional palliative bypass for malignant GI obstruction are just examples of what needs to be investigated. Multicenter trials require standardization of MCA outcome reporting. There is an unmet demand for specialized pediatric MCA devices with verified force calibration for neonatal bowel calibers.

## Limitations

### This systematic review has several key limitations

The available clinical evidence remains limited, with only 106 patients reported across 13 studies. A majority of studies were either case reports, e-case reports or small single-center series, which limit the ability to draw definitive conclusions regarding efficacy and safety. There is substantial heterogeneity in device design, magnet strength and procedural technique, making direct comparisons between studies difficult. The variations in indications, devices, and standards for reporting outcomes make quantitative meta-analysis impossible. The duration of follow-up varies from short-term to approximately 3 years, but long-term outcomes are mostly unavailable. Finally, publication bias may exist, as unsuccessful cases or complications may be underreported.

## Conclusion

With zero anastomotic leakage seen in all 13 included trials and 106 patients representing a variety of indications, institutions, and patient groups, magnetic compression anastomosis is a therapeutically adaptable, safe, and technically advanced minimally invasive approach. In patients with malignant obstruction, MCA without general anesthesia may allow the prompt start of oncological treatment, which may be a significant survival benefit. With no biliary fistula, MCA shortens the most technically challenging anastomoses in laparoscopic pancreatoduodenectomy from 25–57 min to 8–16 min for CJ and 12–15 min for PJ. Swallowable biofragmentable magnets produce metabolic jejunoileal and gastroileal bipartitions during bariatric surgery, resulting in day-case hospital stays and notable improvements in type 2 diabetes. MCA maintains sphincter integrity and produces excellent long-term functional results in pediatric surgery by repairing congenital or acquired rectal atresia without requiring open pelvic dissection. Beyond the gastrointestinal system, magnetic anastomosis technology can be used in vascular surgery to significantly shorten operating times, improve patency rates, prevent smooth muscle cell hyperplasia, and shorten the anhepatic phase in liver transplantation from more than 35 min to less than 13 min. The crucial next step in determining MCA’s definite significance across the entire spectrum of surgical anastomosis is to conduct prospective randomized trials with standardized devices and result reporting.

## Supplementary Information

Below is the link to the electronic supplementary material.

Supplementary file1 (DOCX 20 KB)

Supplementary file2 (DOCX 55 KB)
