# Supplementary material for: Magnetic compression anastomosis in gastrointestinal surgery: a systematic review of early human experience
Source: Surg Endosc. 2026 Jul 16;40(9):7954–66. doi: 10.1007/s00464-026-12956-6 (PMC13582030; doi:10.1007/s00464-026-12956-6)
Supplement: Supplementary file 1 [file 464_2026_12956_MOESM1_ESM.docx]

Supplementary Table S1: Methodological quality assessment

*Magnetic Compression Anastomosis in Gastrointestinal Surgery: A Systematic Review of Early Human Experience*

Kaur A, Shaikh S, Valdivieso Rueda SC, Bokhari F, Mani VR - OSF Saint Francis Medical Center / University of Illinois College of Medicine at Peoria

Part A: MINORS quality assessment — cohort and prospective studies (n = 7)

MINORS (Methodological Index for Non-Randomized Studies) scoring: 0 = not reported; 1 = reported but inadequate; 2 = reported and adequate. Maximum score = 16 (non-comparative design). Case reports (n = 6) are not scored by MINORS; CARE assessment is provided in Part B.

| **Study** | **1. Stated aim** | **2. Consecutive pts** | **3. Prospective data** | **4. Endpoints approp.** | **5. Unbiased assess.** | **6. Follow-up approp.** | **7. Loss to f/u <5%** | **8. Sample size calc.** | **Total /16** | **Quality** |
| --- | --- | --- | --- | --- | --- | --- | --- | --- | --- | --- |
| **Prospective first-in-human trials (n = 5)** | | | | | | | | | | |
| **Graves et al. 2017**  *Prospective FIH · USA* | **2** | **2** | **2** | **2** | 1 | 1 | **2** | 0 | **12** | Moderate |
| **Gagner, Fried et al. 2025 (MagJI)**  *Prospective FIH · Czech Republic/Canada* | **2** | **2** | **2** | **2** | 1 | 1 | **2** | 0 | **12** | Moderate |
| **Gagner et al. 2025 (MagGI)**  *Prospective FIH · Canada* | **2** | **2** | **2** | **2** | 1 | 1 | **2** | 0 | **12** | Moderate |
| **Gagner et al. 2024 (multicenter MagDI)**  *Prospective multicenter · Canada/Belgium/Spain/Georgia* | **2** | **2** | **2** | **2** | 1 | 1 | **2** | 0 | **12** | Moderate |
| **Gagner et al. 2025 BMAS (solo MagDI)**  *Prospective FIH · Canada/Georgia* | **2** | **2** | **2** | **2** | 1 | 1 | **2** | 0 | **12** | Moderate |
| **Retrospective cohort studies (n = 2)** | | | | | | | | | | |
| **Kamada et al. 2021**  *Retrospective cohort · Japan* | **2** | 1 | 0 | **2** | 1 | **2** | **2** | 0 | **10** | Moderate |
| **Li, Lv et al. 2021**  *Retrospective cohort · China* | **2** | 1 | 0 | **2** | 1 | 1 | **2** | 0 | **9** | Moderate |
| **Maximum possible score** | **2** | **2** | **2** | **2** | **2** | **2** | **2** | **2** | **16** |  |

**Item 1.** Clearly stated aim: all prospective studies scored 2; purpose and population stated explicitly.

**Item 2.** Inclusion of consecutive patients: 2 for prospective studies (consecutive enrolment explicitly stated); 1 for retrospective studies (consecutive enrolment not confirmed).

**Item 3.** Prospective data collection: 2 for prospective studies by design; 0 for retrospective studies.

**Item 4.** Endpoints appropriate to aim: 2 for all studies — primary endpoints (anastomotic leakage, technical success) directly address stated aims.

**Item 5.** Unbiased endpoint assessment: 1 for all studies — no blinded outcome adjudication reported in any included study; all assessments were by the treating surgical team. Limitation acknowledged.

**Item 6.** Follow-up period appropriate to study aim: 2 where follow-up duration exceeds the expected time to anastomotic healing or clinical stability; 1 where follow-up is short relative to the indication (e.g. oncology or metabolic outcomes requiring longer observation).

**Item 7.** Loss to follow-up <5%: 2 for all cohorts where all enrolled patients are accounted for in outcome reporting.

**Item 8.** Prospective calculation of study size: 0 for all studies. No included study reported a formal power calculation. This is expected for first-in-human exploratory device trials and does not represent a critical methodological failure, but remains a MINORS limitation requiring acknowledgment.

**Score interpretation:** Good ≥12/16; Moderate 9–11/16; Poor ≤8/16.

Part B: CARE quality assessment — case reports (n = 6)

CARE (CAse REport) guidelines scoring applied to six individual case reports. Items scored: 0 = not reported; 1 = reported but incomplete; 2 = reported and adequate. CARE item 7 (patient perspective) was universally absent — none of the six case reports documented the patient's own reported experience or preferences.

| **Study** | **Clinical history & timeline** | **Diagnostic evaluation** | **Intervention details** | **Follow-up & outcomes** | **Patient perspective** | **Informed consent stated** | **CARE rating** |
| --- | --- | --- | --- | --- | --- | --- | --- |
| **Kamada et al. 2020**  *Case report · Japan* | **2** | **2** | **2** | 1 | 0 | **2** | Adequate |
| **Liu et al. 2020**  *Case report · China* | **2** | **2** | **2** | **2** | 0 | **2** | **Good** |
| **Lu, He et al. 2021**  *E-video · China* | **2** | 1 | **2** | 1 | 0 | **2** | Adequate |
| **Zhang et al. 2023**  *E-video · China* | **2** | 1 | **2** | 1 | 0 | **2** | Adequate |
| **Tyraskis et al. 2025**  *Case report · UK* | **2** | **2** | **2** | **2** | 0 | **2** | **Good** |
| **Zhang et al. 2024**  *E-video · China* | **2** | 1 | **2** | 1 | 0 | **2** | Adequate |

**Column definitions:** Clinical history & timeline = CARE items 4–5; Diagnostic evaluation = CARE item 6; Intervention details = CARE item 8; Follow-up & outcomes = CARE item 9; Patient perspective = CARE item 10; Informed consent stated = CARE item 3.

**Patient perspective (0 in all cases):** None of the six case reports document the patient's own narrative or reported experience. This is a consistent gap across surgical case report literature but is a required CARE item.

**CARE rating:** Good = 5–6 items adequate; Adequate = 3–4 items adequate; Poor = ≤2 items adequate.
