# Supplementary material for: Magnetic compression anastomosis in gastrointestinal surgery: a systematic review of early human experience
Source: Surg Endosc. 2026 Jul 16;40(9):7954–66. doi: 10.1007/s00464-026-12956-6 (PMC13582030; doi:10.1007/s00464-026-12956-6)
Supplement: Supplementary file 2 [file 464_2026_12956_MOESM2_ESM.docx]

**Identification of studies via databases and registers**

Records removed *before screening*:

Duplicate records removed (n =54 )

Records marked as ineligible by automation tools (n = 0)

Records removed for other reasons (n = 0)

Records identified from*:

Databases (n = 268)

Registers (n = 0)

**Identification**

Records screened

(n = 209)

Records excluded**

(n =192)

Reports sought for retrieval

(n =17 )

Reports not retrieved

(n = 0)

**Screening**

Reports excluded:

Preclinical/animal studies (n = 1)

Conference abstract only (n = 1)

Editorial/commentary/non-original study (n = 1)

• No relevant clinical outcomes reported (n = 1)

Reports assessed for eligibility

(n =17 )

Reports of included studies

(n = 13)

**Included**

*Consider, if feasible to do so, reporting the number of records identified from each database or register searched (rather than the total number across all databases/registers).

**If automation tools were used, indicate how many records were excluded by a human and how many were excluded by automation tools.

Source: Page MJ, et al. BMJ 2021;372:n71. doi: 10.1136/bmj.n71.

This work is licensed under CC BY 4.0. To view a copy of this license, visit <https://creativecommons.org/licenses/by/4.0/>
